# Unraveling the ecological processes modulating the population structure of *Escherichia coli* in a highly polluted urban stream network

**DOI:** 10.1038/s41598-021-94198-1

**Published:** 2021-07-19

**Authors:** Martín Saraceno, Sebastián Gómez Lugo, Nicolás Ortiz, Bárbara M. Gómez, Carmen A. Sabio y García, Nicolás Frankel, Martín Graziano

**Affiliations:** 1 Instituto de Ecología, Genética y Evolución de Buenos Aires (IEGEBA), CONICET - Universidad de Buenos Aires, 1428 Buenos Aires, Argentina; 2 Departamento de Ecología, Genética y Evolución, Facultad de Ciencias Exactas y Naturales, Universidad de Buenos Aires, 1428 Buenos Aires, Argentina; 3 Instituto Nacional del Agua, 1804 Ezeiza, Argentina; 4 Instituto de Fisiología, Biología Molecular y Neurociencias (IFIBYNE), CONICET - Universidad de Buenos Aires, 1428 Buenos Aires, Argentina

**Keywords:** Microbial ecology, Limnology, Urban ecology

## Abstract

*Escherichia coli* dynamics in urban watersheds are affected by a complex balance among external inputs, niche modulation and genetic variability. To explore the ecological processes influencing *E. coli* spatial patterns, we analyzed its abundance and phylogenetic structure in water samples from a stream network with heterogeneous urban infrastructure and environmental conditions. Our results showed that environmental and infrastructure variables, such as macrophyte coverage, DIN and sewerage density, mostly explained *E. coli* abundance. Moreover, main generalist phylogroups A and B1 were found in high proportion, which, together with an observed negative relationship between *E. coli* abundance and phylogroup diversity, suggests that their dominance might be due to competitive exclusion. Lower frequency phylogroups were associated with sites of higher ecological disturbance, mainly involving simplified habitats, higher drainage infrastructure and septic tank density. In addition to the strong negative relationship between phylogroup diversity and dominance, the occurrence of these phylogroups would be associated with increased facilitated dispersal. Nutrients also contributed to explaining phylogroup distribution. Our study proposes the differential contribution of distinct ecological processes to the patterns of *E. coli* in an urban watershed, which is useful for the monitoring and management of fecal pollution.

## Introduction

*Escherichia coli*, one of the most frequently found pathogens in urban waters, has historically been used as a proxy for recent microbiological pollution, mainly due to its straightforward and low-cost detection methods^1^. There is consistent evidence that some *E. coli* strains may persist and even grow in secondary habitats such as fresh and marine waters and sediments^2–6^. The ecological processes influencing the population structure of *E. coli* in urban environments are unclear; its ubiquity result from a complex balance involving external inputs, ecological conditions and niche processes acting on aquatic habitats, while affecting its intraspecific genetic variability^7–9^. Therefore, a better understanding of the ecological processes underlying the environmental occurrence of *E. coli* is a matter of methodological, sanitary and management concern^9,10^.

*E. coli* intra-specific variability has been grouped into eight major phylogroups based on genetic analyses: A, B1, B2, C, D, E, F and G^11–13^. Although laboratory and pathogenic *E. coli* strains have been thoroughly described, the environmental ecological niches associated with phylogroups remain largely unknown. The abundance pattern of each phylogroup seems to vary among surface freshwater systems, but some of them (A and B1) are consistently predominant and prevalent while others are less frequent and associated to urban areas (e.g., D and F)^14–16^. According to the coexistence theory, the over-representation of some phylogroups in the environment can be ascribed to competitive fitness differences (e.g., in resource exploitation) that stabilize their coexistence^17^. Experimental microcosm studies support the notion that the most abundant *E. coli* phylogroups show greater capacity of environmental survival. For example, strains from phylogroup B1 were found to persist longer and to tolerate lower temperatures than the remaining phylogroups^18^. Moreover, phenotypic analysis revealed that phylogroup B1 strains were more likely to exhibit traits indicative of a higher ability to colonize aquatic plants (and therefore to persist in freshwater secondary habitats), while A and B2 phenotypes were linked to an animal-associated lifestyle^19^. *Escherichia* strains closely related to *E. coli* (i.e., *cryptic clades* I to V), were also associated with long-term environmental persistence^3,20,21^. Altogether, this strongly suggests that niche processes such as competitive exclusion and niche partitioning may contribute to shaping the spatial distribution of the environmental population structure of *E. coli*. However, there is no *in-situ* evidence supporting the role of niche processes.

Several studies focused on bacterial community in urban streams have revealed the impact of urbanization on population structure, highlighting the need to consider watersheds as part of a complex social-ecological-technological system^22–24^. Within this framework, ecological modulations may be understood as a balance between local features of the ecosystems, demographic pressures and built-in infrastructure. In this sense, urban infrastructures, such as stormwater networks and roads, facilitate the transfer of pollutants by surface runoff, acting as links between human activity in land areas of the watershed and water bodies, turning the latter into sinks^25^. Indeed, the presence of drainage infrastructures and impervious surfaces was positively associated with concentrations of dissolved organic carbon (DOC), soluble reactive phosphorus (SRP), total phosphorus (TP), ammonium, nitrates and nitrites, and electrical conductivity in urban streams^26–28^. Some of these compounds can be used as nutrients by aquatic bacteria^8,29^. Thus, heavily impacted urban water bodies with high availability of these nutrients and other suitable environmental conditions (e.g., relatively high temperatures and a wide range of colonizable niches) may become hot habitats for *E. coli* strains showing long-term persistence^30–33^.

Previous studies have suggested that environmental persistence of *E. coli* in urban freshwater systems is influenced by abiotic factors such as soil moisture, temperature and nutrient availability^7,34^. Furthermore, it was shown that high levels of soluble nutrients (e.g., DOC and phosphorus) have a positive influence on growth and survival of *E. coli* in the water column of urban streams^8^. Moreover, the input of black and grey waters into streams entails the dispersal of allochthonous bacteria, representing one of the major drivers of shifts in the microbial community composition of these systems^35–37^. Septic tanks are also known to contribute to non-point pollution through leakage, acting as a source of pathogens, dissolved nutrients and metals to groundwater and nearby streams^38,39^. Likewise, sanitary systems such as sewer networks, if present, cause pollution by leakage and subsequent exfiltration to water bodies^39,40^. Hence, ecological niche conditions and human-facilitated dispersal processes may also play an important role in modulating *E. coli* population structure in urban surface waters.

In this work we analyzed the ecological processes affecting the spatial structure of *E. coli* in a heavily polluted urban stream network of Buenos Aires province, Argentina. The watershed was characterized as a social-ecological and technological system in order to analyze the contributions of local urban infrastructure and environmental conditions. Spatial processes structuring *E. coli* populations that were not explained by the latter were assessed by including asymmetric eigenvector maps. We hypothesize that the density of input sources to streams and the features of the ecological habitat determine the spatial patterns of abundance and phylogenetic composition of *E. coli*. In this context, we predict that (1) *E. coli* abundance will be positively associated with the density of different urban input sources and higher nutrient availability, (2) that the occurrence of phylogroup B1 will be associated with higher nutrient availability and aquatic vegetation coverage and (3) that the occurrence of low-frequency *E. coli* phylogroups (D and F) will be positively associated with locations of higher urbanization. Finally, we discuss our results in relation to the different ecological processes that could influence the dynamics of *E. coli* and their implications for urban water management.

## Methods and materials

### Study area and sampling procedure

**Figure 1:**
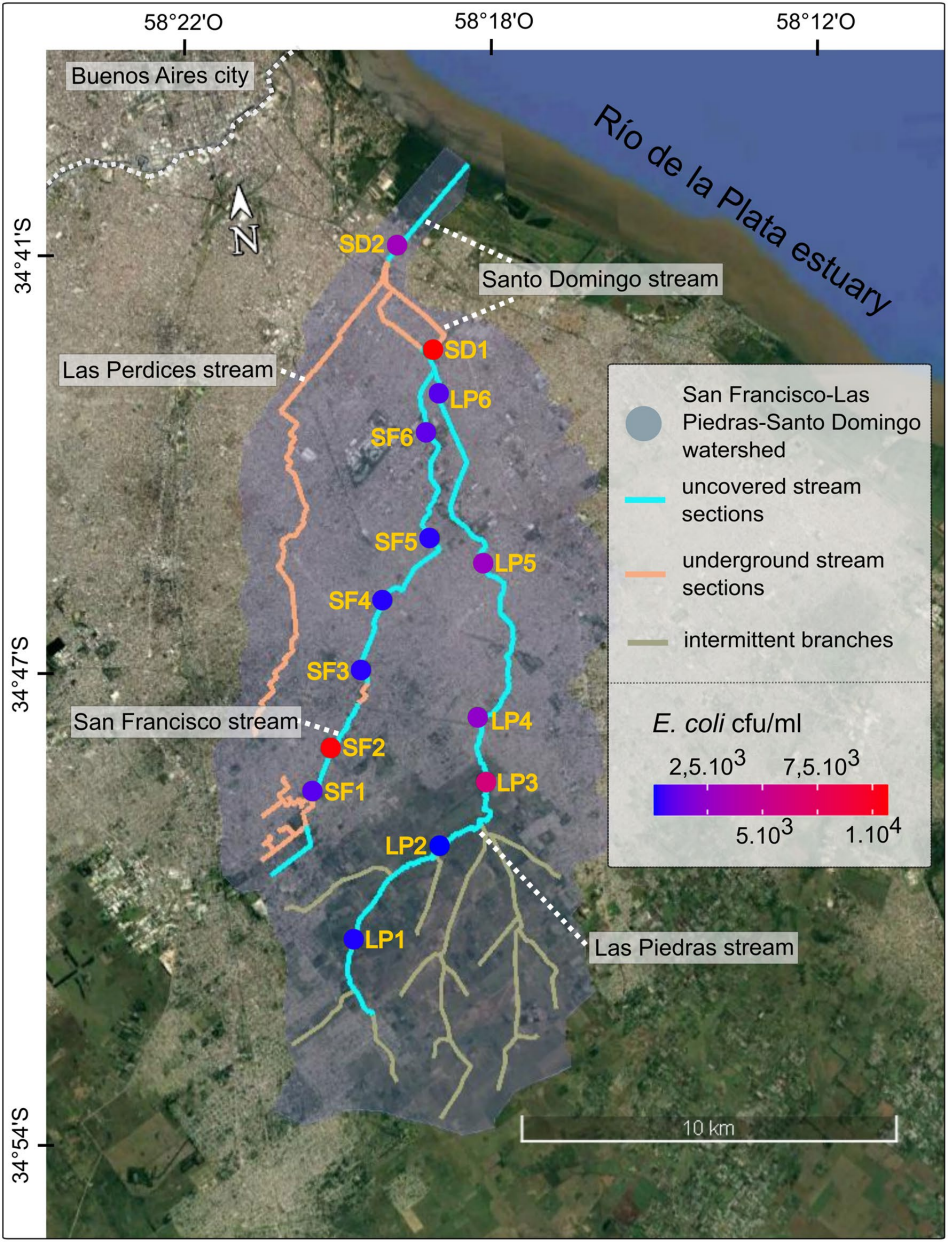
Location of the San Francisco-Las Piedras-Santo Domingo watershed and *E. coli* abundance across sampled sites. The studied watershed is located on the southern side of the Metropolitan Area of Buenos Aires. Spatial distribution of mean *E. coli* abundance (cfu/ml) at the sampled sites (dots) is color-coded in a blue-red gradient. LP, Las Piedras stream; SF, San Francisco stream; SD, Santo Domingo stream. Base map created in Google Earth V 7.3.3.7786. (October 5, 2020). Buenos Aires Metropolitan Area, Argentina. 34°46ʹ47.32ʺS, 58°19ʹ27.95ʺO, Eye alt 47.5 km. Maxar Technologies 2020. http://www.earth.google.com. Graphical edition in Inkscape V 0.92.4. Inkscape Project (2020). https://inkscape.org.

The watershed of San Francisco, Las Piedras and Santo Domingo streams is located in the Pampean plain, in the south of the highly urbanized Buenos Aires Metropolitan Area (AMBA), surrounding Buenos Aires city (Fig. 1). This watershed encompasses a total surface area of approximately 160 km^2^, with a longitudinal extension of about 23 km. The streams draining this area are affected by multiple anthropogenic stressors^41^. The watershed and stream network have been highly modified by rectifications, channel incisions and some streams were partially piped; one of the latter (Las Perdices stream) was even relocated into this watershed. In addition, urban growth in the AMBA was not accompanied by investments in sanitary infrastructure and as a result, local water bodies receive the impact of untreated sewage and domestic effluents^42–44^.

We selected 14 sampling sites across the watershed representing a broad range of urban characteristics related to population density and coverage of sanitary services. Data were obtained from the Census Data Center^45^. The sampling campaign was conducted in summer, the season with the most favorable mean temperature for *E. coli* survival, during 4 consecutive days in February 2018. A dry period of at least 4 days was considered before the sampling camping and no rainfall were recorded during its duration. Sites across the stream network were visited randomly at morning-noon to avoid spatio-temporal dependence. Habitat heterogeneity was assessed along a 50 m-longitudinal transect established along the main watercourse at each sampling site, where sub-samples were taken at 0 m, 25 m and 50 m. On each sub-sampling site, pH, dissolved oxygen (DO), conductivity and temperature (T) were measured in situ from the sub-surface layer of the water column, using a Lutron WA-2017SD multiparameter field sensor. Local hydraulic and habitat parameters of the sampled stream section were assessed in terms of physical dimensions (width and depth), flow velocity and macrophyte coverage. Flow velocity was determined using the flotation method by triplicate and water flow from physical and flow velocity parameters^46^. Mean values of flow velocity, depth and water flow were calculated per site. Macrophyte coverage was estimated according to the Braun-Blanquet methodology, by establishing a 0.5 m width plot perpendicular to the flow and accounting for emerged and floating-leaved macrophytes^47^. Water samples from each sub-sampling site were collected in clean 250-ml bottles for the analysis of dissolved nutrients and other analytes and in sterile 810-ml plastic bags (Hixwer) for measuring microbiological parameters. Water samples were taken from the sub-surface layer and kept cold until processing in the laboratory.

### Physicochemical characterization

Upon arrival at the laboratory, turbidity was measured with a turbidimeter HACH 2100P. Samples were filtered simultaneously for further quantification of soluble analytes. First, samples were filtered through a cellulose nitrate filter of 0.47 µm-pore diameter (Sartorius) for subsequent analysis of soluble reactive phosphorus (SRP), ammonium and nitrates. Then, they were passed through a polycarbonate filter of 0.2 µm-pore diameter (Merck Millipore) for dissolved organic carbon (DOC), soluble iron and chloride. SRP was determined by the molybdovanadate method, ammonium by the salicylate method and nitrate plus nitrite by the cadmium reduction method^48^. Measurements were made by colorimetric analysis with a spectrophotometer Hach® DR 2800 within 48 h of sample collection; limits of quantification (LOQ) were 0.1 mg/L for SRP and nitrates and 0.4 mg/L for ammonium. Ammonia and nitrate plus nitrite were considered together as dissolved inorganic nitrogen (DIN). For DOC determination, filtered samples were acidified with high-quality sulfuric acid (Merck) and measurements were performed with the high-temperature combustion method using a total organic carbon analyzer Shimadzu 5000A; LOQ was 0.1 mg/L^48^. Chlorides were measured by the iodometric titration method; LOQ was 4 mg/L^48^. Finally, filtered samples pre-treated with ultrapure nitric acid (Merck) were analyzed for soluble iron by inductively coupled plasma-mass spectrometry (ICP-MS) using a mass spectrometer Agilent 7500cx; LOQ was 0.3 µg/L.

### *Escherichia coli* abundance and phylogenetic group annotation

*Escherichia coli* abundance was determined for each sample (42 samples in total, three per site) by plate counting using Chromocult® Coliform Agar selective medium (MilliporeSigma). Serial dilutions in sterile ultrapure water were performed on each sample in triplicate and plates were cultured for 24 h at 37 °C. After scoring for blue- to purple-colored colonies in the best dilution for each sample, triplicates counts were averaged per sample and relativized to one milliliter of volume. The detection limit was 200 cfu/100 mL.

To obtain the relative phylogenetic composition of *E. coli* per location, in a first step, blue- to purple-colored colonies from the Chromocult® plates from each site were streaked onto Levine E.M.B. (Eosin Methylene Blue) agar plates and cultured for 24 h at 36 °C. The streaking procedure was repeated at least once under the same culture conditions to ensure the purity of each isolate. Isolates yielding typical responses for *E. coli* on all media were designated *E. coli*, while those exhibiting no typical phenotypic responses in any of the media were further screened using a colony PCR method with primers directed to *E. coli* essential *trp*A gene^49^. A total of 327 isolates were reliably assigned to *E. coli*, ranging from 18 to 33 isolates per sampled site. Isolates were randomly selected from the sub-sampling plates (see Table S1, Supplementary Appendix A1 for total counts per site). Phylogenetic annotation within the *E. coli* intraspecific groups was then performed using the Clermont’s multiplex PCR method^13^. The closely related phylogroups G and F were grouped together because this strategy is unable to differentiate one from the other^12^. Selected isolates were grown overnight in MacConkey broth at 37 °C with continuous shaking and used as templates in the PCR assays. The first round of phylogroup assignment was performed through the amplification of the *ara*A, *chu*A, *yja*A and TspE4.C2 genes. A remarkable advantage of this method is that at least one of the targeted genes is guaranteed to be amplified, thus allowing a positive control of species identity. Additional primers were used to identify phylogroups E and C if needed, by detection of *arp*A and *trp*A genes, respectively. A double PCR method, based on *aes* and *chu*A allele‐specific amplification, was employed to assign an *Escherichia* strain a cryptic lineage membership^50,51^. All PCR reactions were carried out in a 20 µL-total volume containing 2 µL of templates and 10 µL of 2X GoTaq® Green Master Mix (Promega) following the published literature^13,51,52^. The complete assignment of isolates to phylogroups per site is shown in Table S1 (Supplementary Appendix A1).

### Demographic, hydraulic and sanitary infrastructure parameters

A 500-m radius circular area was established around each site to survey the total number of dwellings and residents and the total number of dwellings with sanitary sewers, septic tanks and potable water, using open data provided by national agencies^45^. The impervious surface coverage was calculated through GIS image processing (QGIS v. 2.18.20) coupled with the Semi-automatic Classification Plugin algorithm, employing Landsat 8 satellite images of the watershed^53^. This methodology is based on the preliminary creation of a spectral firm by a supervised classification procedure for the generation of regions of interest (ROIs). Each pixel in the ROI was categorized as impervious or pervious area. The trained ROIs were employed with the Spectral Angle Mapper algorithm to characterize the satellite image of the watershed. Impervious surface coverage was first calculated by manual digitalization of about 30 ha distributed homogeneously throughout the basin, obtained from a Google Maps image ^54^. This was used to generate polygons representing areas with different percentages of imperviousness. Permeable areas showed a limit of impermeability of 5%. Finally, considering a 500-m radius buffer area for each sampling site, GIS processing tools were used to calculate the areas for the two permeability types, to further express the impervious surface as a percentage within each buffer area. To estimate drainage and road densities, layouts of the pluvial and road networks were provided by local and provincial administrations agencies. Data were intersected with the buffer areas to calculate the length of their respective pluvial and road sections.

### Estimation of spatial predictors

Asymmetric Eigenvector Maps (AEMs) were generated to model directional spatial processes underlying streams network singularities^54–56^. These AEMs were based on the geographical distribution of the sampling sites, and the connectivity matrix among sites was constructed considering the upstream–downstream flux and a maximum spatial neighborhood distance of 5 km between sites. The AEMs also included weight vectors obtained from the connectivity matrix, which determined the connection strength between samples according to their geographic distance. A two-sided Moran’s test (999 permutations; *p* < 0.05) was used to retain AEMs showing significant positive or negative spatial autocorrelations (see Supplementary Fig. S1 for more details)^57^. Finally, *forward selection* procedures were carried out separately for each response variable to retain AEMs significantly associated with them. This yielded 6 and 11 AEMs that were significantly associated with *E. coli* abundance and phylogenetic composition, respectively. The four AEMs gathering the greatest R^2^ contribution were employed to avoid multicollinearity in further analysis involving *E. coli* phylogenetic composition. These procedures were conducted using the packages *adespatial* (v 0.3–8), *ade4* (v 1.7–15), *sp* (v 1.3–2) and *spdep* (v 1.1–3) in R. For more details on the methodology for spatial-predictor selection, see Appendix A2 of the Supplementary Material.

### Data analysis

We first calculated the phylogroup specific diversity and dominance at each site for an initial characterization of the phylogroup population structure^58,59^. Phylogroup diversity was obtained by counting the number of different phylogroups present at each site, while Simpson’s dominance index (D) was calculated as the weighted arithmetic mean of their proportional abundances^58^. The association between phylogroup diversity, dominance and *E. coli* mean abundance was analyzed using a Pearson’s correlation analysis.

Different statistical approaches were applied to analyze relationships among predictors. Initially, two sets of predictor variables were established: an environmental matrix including local physicochemical and habitat conditions and an urban infrastructure set, combining demographic information with different aspects of sanitary infrastructure at the watershed scale. A Principal Component Analysis (PCA) was performed to analyze the association between sites and environmental and urban infrastructure variables^60^. Additionally, a Pearson’s correlation matrix with hierarchical clustering was performed among variables. The significance of the correlation coefficients (Pearson’s ρ) was evaluated with the paired-samples correlation test adjusted for multiple comparisons (Holm’s method)^61^. In addition, we analyzed the network of global correlations between pairs of variables showing an absolute Pearson’s ρ greater than 0.5. To understand the importance of each parameter in determining the whole network’s structure, we estimated associated centrality measures such as Expected Influence (EI), which represents the strength of a node’s influence within the network^62^.

A variation partitioning analysis was performed to disentangle the pure and shared effects of the local environment, urban infrastructure and spatial predictors determining the patterns of abundance and phylogroup composition (based on presence-absence data) of *E. coli*^63^. An adjusted multivariate redundancy statistic (R_a_^2^) was used to analyze the proportion of variance explained by each component. To reduce the number of variables and to avoid multicollinearity between predictors, a set of multivariate analyses were performed. This was accomplished by identifying several groups of covariate variables by means of a PCA analysis applied to the environmental and infrastructure matrices separately, along with the use of variance inflation factors (for more details on the procedure for variable selection, see Appendix A3 of the Supplementary Material).

For variance partitioning and partial redundancy analyzes, the *E. coli* abundance and the predictor matrices were previously standardized by the *Standardization* method^64^. In accordance with Leps & Smilauer^65^, we preliminarily assessed the use of a linear constrained model (RDA) onto phylogroup composition data, by considering the length of the gradient obtained with a Detrended Correspondence Analysis (DCA; 1st axis length = 1.44). The significance of global models and variables was further evaluated through partial redundancy analysis (pRDA), which was subjected to different conditioning constraints. In the case of the *E. coli* abundance, two different constraints were implemented (1) a pRDA analysis of the environmental or infrastructure matrices, conditioned by each other, and (2) a pRDA analysis of spatial predictors conditioned by the rest of the predictor sets. In contrast, for the occurrence of *E. coli* phylogroups, each predictor matrix was conditioned by the rest of the variables. In all cases, statistical significance of pRDA was tested using a restricted Monte Carlo Permutation Test of the residuals of the full model (9999 permutations), to account for our nested sampling design and the multilevel structure of the explanatory variables^66^. Both normality and lack of structure in residuals were tested. Statistical procedures were implemented with the packages *vegan* (version 2.5–6), *corrplot* (version 0.84), *qgraph* (version 1.6.5) and *permute* (version 0.9–5) in R.

## Results

### Patterns of *E. coli* abundance associated with environmental and urban infrastructure features

To gain insight into the ecological processes modulating the population structure of *E. coli*, we assessed the joint and independent contributions of urban features and local stream habitat conditions across 14 stream reaches (locations) distributed throughout a heavily polluted urban stream network with heterogeneous built-in sanitation and drainage infrastructure (Fig. 1). The characterization of urban features included demographic, hydraulic (impervious surfaces, drainage density and road density) and sanitary conditions (sanitary sewers, septic tanks and potable water) (Table 1). Local habitats were characterized for their physicochemical profile (pH, conductivity, temperature, dissolved oxygen, DIN, SRP, DOC, turbidity, chlorides and iron) and for local hydraulic and habitat parameters of the stream section (physical dimensions, water flow, flow velocity and macrophyte coverage) (Table 1). Spatial predictors were also included in the analysis to account for directional and correlated effects (see Appendix A2 of the Supplementary Material). *E. coli* abundance was quantified at each location and the phylogenetic affiliation of isolates was assigned by molecular methods.

**Table 1:** Basic statistical summary of environmental (unbolded) and urban infrastructure measured parameters (bolded).

	Range	Mean	SD
pH	5.27–8.02	7.36	0.74
Conductivity (μS/cm)	762–1533	1078	189
Temperature (°C)	17.4–23.1	19.6	1.7
Dissolved oxygen (mg/L)	2.5–10.0	4.1	2.0
DIN (mg/L N-NO_3_^−^)	5.6–20.0	12.4	4.3
SRP (mg/L P-PO_4_^3−^)	0.8–7.3	2.3	1.7
DOC (mg/L)	6.2–77.9	18.0	18.0
Turbidity (NTU)	6–109	34	33
Chloride (mg/L)	38–107	70	22
Iron (μg/L)	23.5–337	135	91.6
Macrophyte coverage (%)	0–92	38	31
Water flow (m^3^/s)	3.0 × 10^–2^–3.8	0.9	1.2
Flow velocity (m/s)	3.21 × 10^−2^–0.43	0.21	0.12
Depth (cm)	28–80	46	16
**Drinking water coverage (%)**	**0.01–31.1**	**13.4**	**12.5**
**Sanitary sewer density (dwelling/ha)**	**0.0–16.2**	**3.5**	**5.4**
**Septic tank density (dwelling/ha)**	**0.1–28.9**	**14.0**	**9.9**
**Impervious surface (%)**	**5–75**	**53**	**27**
**Drainage density (m/km**^**2**^**)**	**0–3.837**	**1.519**	**1.235**
**Road density (m/km**^**2**^**)**	**1.248–22.369**	**13.983**	**7.323**
**Population density (Hab./ha)**	**0.3–135.0**	**68.7**	**45.4**
*E. coli* (cfu/ml)	3–10,700	2735	3732

DIN, dissolved inorganic nitrogen; SRP, soluble reactive phosphorus; DOC, dissolved organic carbon; *SD*, standard deviation.

**Figure 2:**
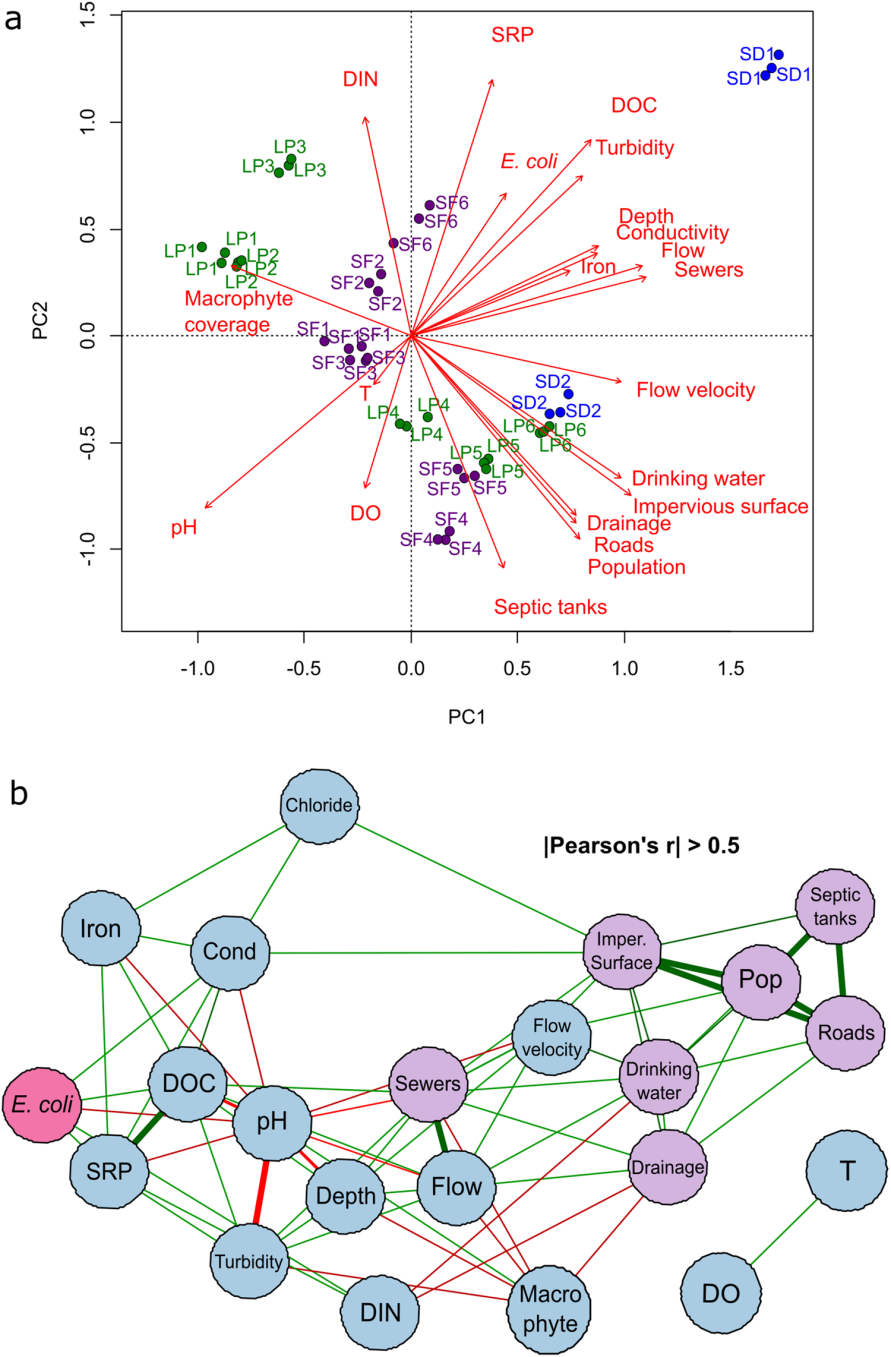
Relationship of *E. coli* abundance with environmental and urban infrastructure features (**a**) Biplot of a principal component analysis including all samples and variables. Sampled sites from San Francisco stream are in purple, Las Piedras stream in green and Santo Domingo stream in blue; (**b**) Network diagram of correlations with an absolute Pearson’s ρ greater than 0.5. Green edges correspond to positive correlations and red edges to negative ones; The edge width indicates the magnitude of Pearson's ρ. Light-blue circles represent the environmental parameters, purple circles the infrastructure variables and pink circles the abundance of *E. coli*. LP, Las Piedras stream; SF, San Francisco stream; SD, Santo Domingo stream; DIN, dissolved inorganic nitrogen; SRP, soluble reactive phosphorus; DOC, dissolved organic carbon; DO, dissolved oxygen; T, temperature; Pop, population density; Cond, conductivity. Data analysis performed in R software^73^. Graphical edition in Inkscape V 0.92.4. Inkscape Project (2020). https://inkscape.org.

The locations surveyed had a wide variability in environmental and urban infrastructure across the hydrological network (Table 1 and Fig. 1). *E. coli* abundance exceeded the level recommended by the US-EPA 2012^1^ of 235 cfu/100 mL for recreational freshwaters at all studied sites, which poses a great risk for people in direct contact with the studied streams (see Table S1, Appendix A1 of the Supplementary Material for detailed results). The analysis of urbanization proxies (infrastructure coverage, impervious surface coverage and population density) allowed to characterize the watershed as predominantly urban (mean impervious surface of 53%), with a low-urbanized area located in the headwater of Las Piedras stream. The rest of the basin is highly urbanized and values of impervious surface are higher downstream (> 70%). However, the coverage of sanitary infrastructure services, such as drinking water or sewerage, is heterogeneous across the watershed, reflecting differences in the level and quality of urbanization. A PCA revealed a gradient of urban infrastructure along the watershed (Fig. 2a) and grouped locations into distinct clusters based on their features. A first cluster includes locations from the upper section of Las Piedras stream, with higher macrophyte coverage and DIN, together with lower infrastructure coverage. A second cluster groups locations from the upper section of the San Francisco stream, which mainly show intermediate infrastructure coverage. Lastly, a third cluster is composed of locations from densely populated areas with a high development of hydraulic and sanitary infrastructure, which are associated with lower levels of nutrients and high flow velocity. One location in the Santo Domingo stream (SD1) was not grouped in any of the clusters identified, mainly associated with elevated levels of DOC, *E. coli* abundance and turbidity.

A network of global Pearson’s correlations was used to explore co-variation patterns between variables (see Figure S4, Supplementary Appendix A4 for the overall significance analysis). The obtained network was analyzed in terms of the influence or relative importance of each variable (see Fig. 2b and Table S4 in Appendix A4 of the Supplementary Material for full-network metrics). Urban infrastructure variables were ordered close to each other by their positive and significant correlations, mainly interacting through the proportion of impervious surface (expected influence, |EI|= 4.80), drinking water coverage (|EI|= 3.65) and population density (|EI|= 3.45). Among environmental variables, flow velocity (|EI|= 2.48), water flow (|EI|= 2.46), and with lower relative influence, macrophyte coverage (|EI|= 1.91) concentrated the links with urban infrastructure, mainly interacting with sanitary sewer density (|EI|= 2.95) and impervious surface. A second branch of interactions was evidenced between impervious surface and conductivity (|EI|= 2.54) and chlorides (|EI|= 1.52). Moreover, pH (|EI|= 4.82) and DOC (|EI|= 3.60) represented key variables within the environmental matrix, gathering the largest number of links. Local environmental variables such as DOC, SRP, Iron, conductivity, chlorides and DIN were also clustered in a second positive and significant co-variation group. *E. coli* abundance was significantly and positively associated with nutrients such as SRP and DOC, and with physicochemical conditions such as pH (−) and conductivity (+), suggesting that urbanization parameters may indirectly influence *E. coli* abundance through environmental factors. Finally, a cluster of strong positive and negative significant correlations was found among sanitary sewer density, pH, water flow, turbidity, depth and macrophyte coverage.

### Spatial distribution of *E. coli* phylogenetic groups

**Figure 3:**
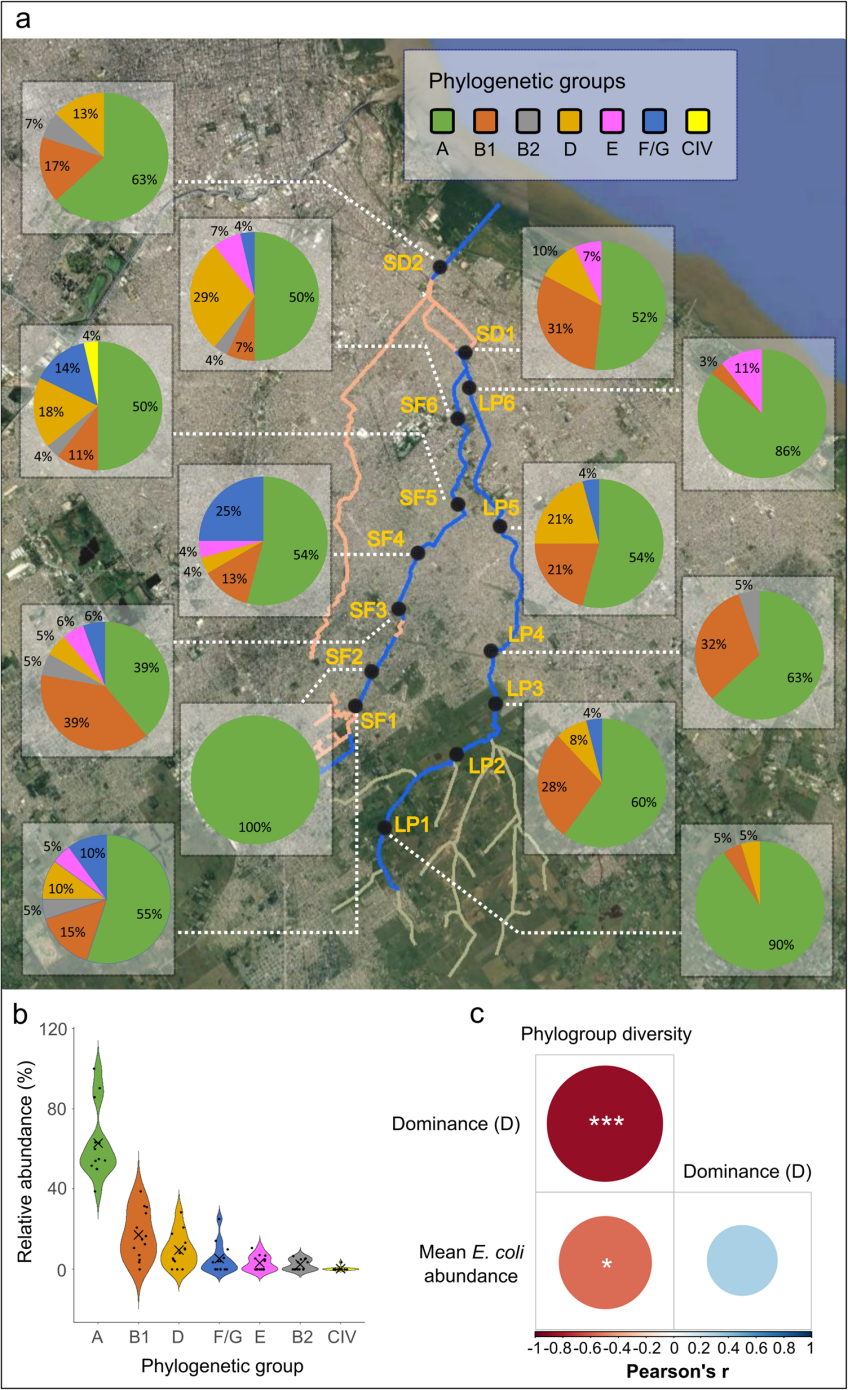
Differences in phylogroup composition of *E. coli* among locations. (**a**) Spatial distribution of the phylogenetic composition in the sampled locations; pie charts indicating the relative frequency of the phylogenetic groups; (**b**) Violin plot of the relative abundance of the phylogenetic groups. Filled dots represent the relative abundance at each site; crosses indicate the means; (**c**) Correlogram of Pearson’s correlations between Simpson’s dominance index (D), diversity of *E. coli* phylogroups, and mean *E. coli* abundance. Significance levels of Pearson’s correlation: *p < 0.05, **p < 0.001. Base map created in Google Earth V 7.3.3.7786. (October 5, 2020). Buenos Aires Metropolitan Area, Argentina. 34°46ʹ47.32ʺS, 58°19ʹ27.95ʺO, Eye alt 47.5 km. Maxar Technologies 2020. http://www.earth.google.com. Data analysis performed in R software^73^. Graphical edition in Inkscape V 0.92.4. Inkscape Project (2020). https://inkscape.org.

A total of 327 environmental isolates were characterized based on the Clermont's multiplex PCR method for *E. coli* phylogroup annotation (see Table S1, Supplementary Appendix A1 for the absolute abundances detected per site). We detected most of the phylogenetic groups, except for phylogroup C. Phylogroup A was the most abundant in all locations sampled, with a relative frequency of up to 50% at most sites, followed by phylogroup B1 (Fig. 3). Mean relative frequency was 64% for phylogroup A, 16% for B1, 10% for D, 5% for F/G, 3% for E and 2% for B2 (Fig. 3b). Notably, a single isolate of the *cryptic clade* IV (further confirmed by a specific multiplex PCR assay for the identification of cryptic clades within the genus *Escherichia*) was collected in location SF5. To our knowledge, this is the first report of a cryptic clade member in surface waters of South America.

A correlation analysis of community structure metrics applied to *E. coli* phylogenetic composition showed that phylogroup diversity was negatively correlated with total mean abundance of *E. coli* (coefficient of correlation ρ =  − 0.56; *p* = 0.05) (Fig. 3c). In addition, Simpson’s dominance index (D) was negatively correlated with phylogroup diversity (coefficient of correlation ρ − 0.87; *p* = 1.10^−4^).

### Disentangling the effects of environmental, urban and spatial factors on patterns of *E. coli* abundance and phylogenetic composition

**Figure 4:**
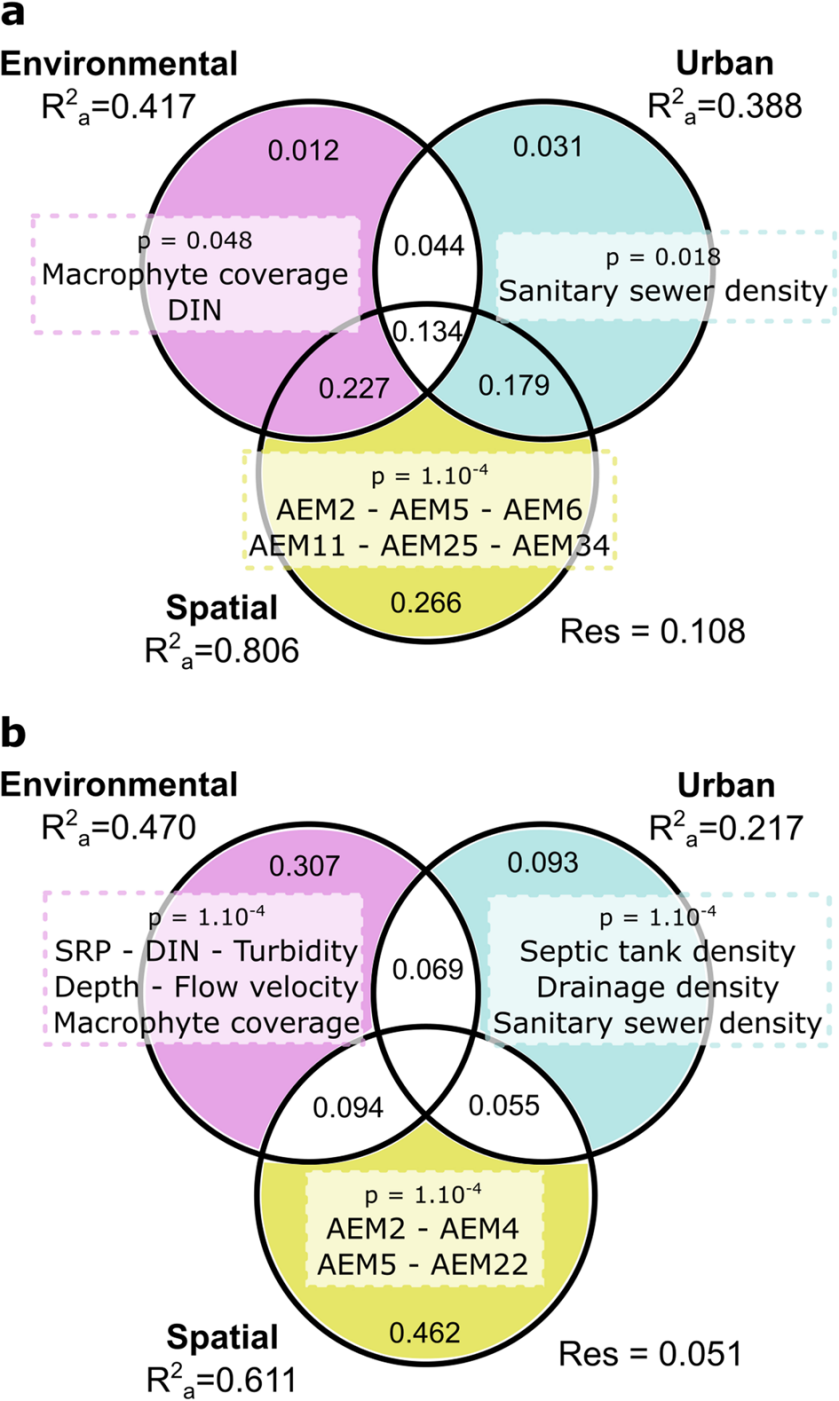
Drivers of *E. coli* population structure. Venn diagrams of the results of variation partitioning analysis of response variables explained by environmental, urban infrastructure and spatial contributing factors: (**a**) abundance of *E. coli*; (**b**) phylogenetic composition. The contribution of each predictor set is represented by R^2^ adjusted values and *p*-values, when available; values < 0 are not shown. Significant parameters derived from each pRDA from each predictor matrix are listed in each diagram (*p* < 0.05); color-shaded areas represent the evaluated fraction for each main matrix. DIN, dissolved inorganic nitrogen; SRP, soluble reactive phosphorus; AEM, asymmetric eigenvector map; Res, residuals. Data analysis performed in R software^73^. Graphical edition in Inkscape V 0.92.4. Inkscape Project (2020). https://inkscape.org.

A variance partition analysis (Fig. 4) showed that important independent effects of environmental and urban infrastructure predictors (23% and 18% of the fraction shared with the spatial matrix, respectively) affect the distribution of *E. coli* abundance. At the same time, the three predictor sets together explained 13% of the total variance. The spatial matrix of AEMs showed a strong pure contribution (27%), while environmental and infrastructure matrices exhibited remarkably lower pure contributions (< 3%) (Fig. 4a). In terms of overall influence, 42% of total contribution to the explained variance was related to the environmental matrix and 39% to the urban infrastructure matrix, indicating a similar contribution of both sets of predictors to the observed patterns of *E. coli* abundance. The high contribution of spatial factors in shared and pure fractions indicates that *E. coli* abundance has a strong directional spatial structure, potentially reflecting distinct spatially structured processes (i.e., different hydrological, environmental and infrastructure patterns).

To further assess the contribution of spatially structured environmental and urban infrastructure variables, a pRDA was performed, where each explanatory set was conditioned by the other (Fig. 4a). Results showed that both environmental (F_7,31_ = 4.48, *P* < 0.05) and urban (F_3,31_ = 7.38, *P* < 0.05) matrices were significant in explaining *E. coli* abundance. Within the environmental set, significant variables were macrophyte coverage (score = 0.43; F_1,31_ = 12.38, *P* < 0.05) and DIN (score = 0.43; F_1,31_ = 12.81, *P* < 0.05). In regard to the urban infrastructure matrix, sanitary sewer density had a significant contribution (score = 0.51; F_1,31_ = 18.02, *P* < 0.05). As well, several coarse- and fine-grade AEMs of the spatial matrix had significant pure contributions (Fig. 4a; see more details in Appendix A2 of the Supplementary Material, Table S2).

**Figure 5:**
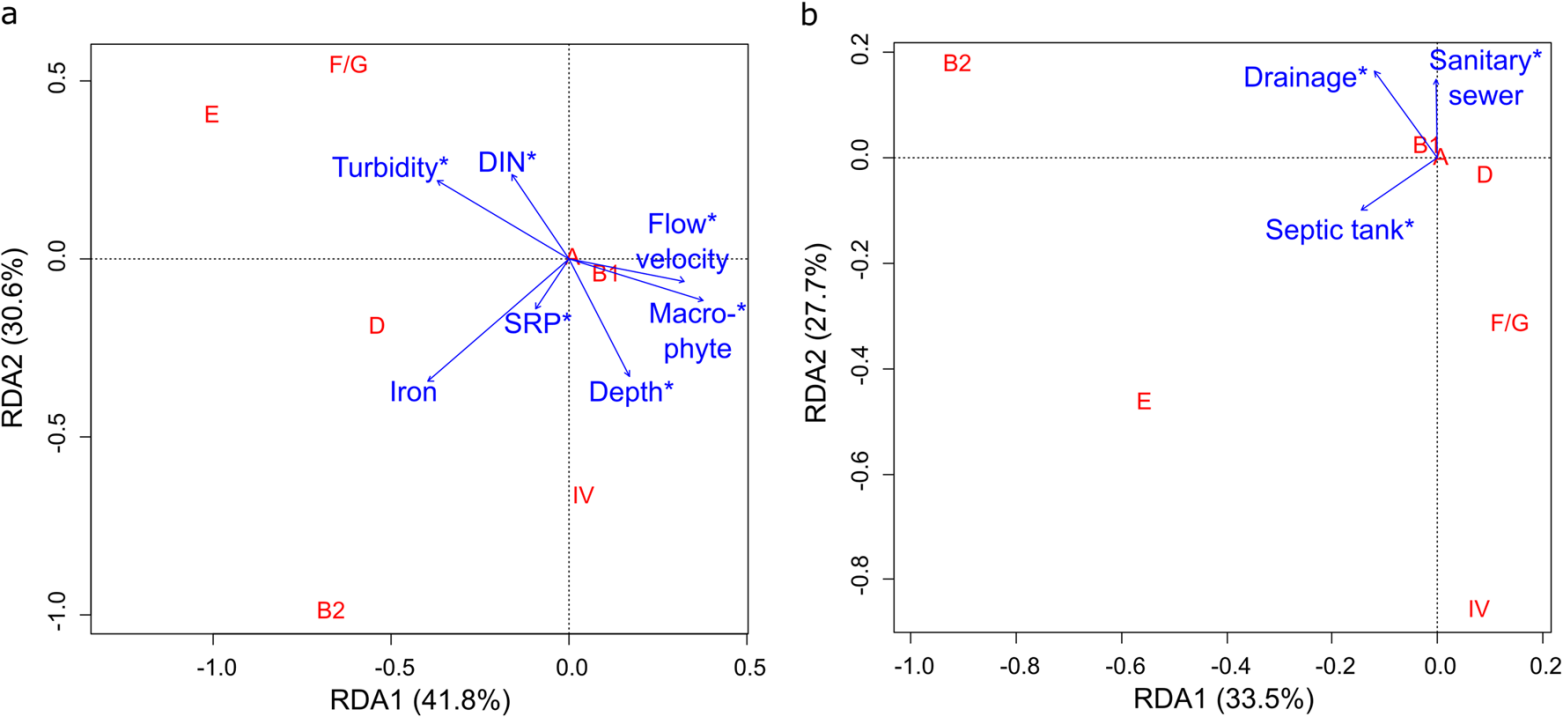
Independent effects of urban infrastructure and ecological habitat conditions affecting the composition of *E. coli* phylogroups. Biplots of partial redundancy analysis relating the occurrence of *E. coli* phylogroups to: (**a**) environmental and (**b**) urban infrastructure explanatory factors (scaling = 3). In each case, pRDA was performed controlling for the rest of the variable sets (including spatial effects). Significant variables, assessed using the Monte Carlo restricted permutation test (9999 permutations), are identified with an asterisk. DIN, dissolved inorganic nitrogen; SRP, soluble reactive phosphorus. Data analysis performed in R software^73^. Graphical edition in Inkscape V 0.92.4. Inkscape Project (2020). https://inkscape.org.

In contrast to the results obtained for abundance, spatial variability in phylogroup composition at the watershed (based on phylogroup presence-absence data) was mostly explained by pure contributions of the three matrices (Fig. 4b). The contribution of spatial factors was the largest (46%), followed by environmental (31%) and urban infrastructure factors (9%). Partial RDA of environmental variables, controlling for the effect of urban infrastructure and spatial AEMs, was statistically significant (F_7,24_ = 27.69, *P* < 0.05). The following variables were significant: DIN (F_1,24_ = 25.95, *P* < 0.05), turbidity (F_1,24_ = 22.99, *P* < 0.05), SRP (F_1,24_ = 20.41, *P* < 0.05), depth (F_1,24_ = 9.03, *P* < 0.05), flow velocity (F_1,24_ = 13.33, *P* < 0.05) and macrophytes coverage (F_1,24_ = 7.04, *P* < 0.05) (Fig. 4b). The first two pRDA axes were also found to be significant: RDA1 accounted for an explained proportion of 42% (F_1,26_ = 98.55, *P* < 0.05) and RDA2 of 31% (F_1,26_ = 72.29, *P* < 0.05). RDA1 was mainly positively related to macrophyte coverage (score = 0.29), flow velocity (score = 0.25) and depth (score = 0.13), and negatively related to turbidity (score =  − 0.29) and potential nutrients DIN (score =  − 0.13) and SRP (score =  − 0.07) (Fig. 5a), while RDA2 was positively related to DIN (score = 0.20) and turbidity (score = 0.19), and negatively associated to depth (score =  − 0.28), SRP (score =  − 0.12) and macrophyte coverage (score =  − 0.10). Phylogroups D, E, F/G and B2 were strongly and negatively correlated with RDA1 suggesting their association with turbidity and higher nutrients conditions, while B1 was slightly and positively associated with it, thus distinguishing between dominant and less frequent phylogroups.

In addition, partial RDA of urban infrastructure predictors, controlling for the effect of environmental and spatial variables was statistically significant (F_3,24_ = 17.39, *P* < 0.05), with a significant association of phylogroup composition with drainage density (F_1,24_ = 11.49, *P* < 0.05), septic tanks density (F_1,24_ = 9.12, *P* < 0.05) and sanitary sewers density (F_1,24_ = 7.27, *P* < 0.05) (Fig. 4b). Significant axis RDA1 (F_1,24_ = 25.53, *P* < 0.05), with an explained proportion of 34%, was negatively related to septic tank density (score =  − 0.29) and drainage density (score =  − 0.24). Moreover, phylogroups B2, E and, to a lesser extent, B1 were negatively associated with RDA1, while F/G and D were positively associated with this axis (Fig. 5b). Axis RDA2, which accounted for 28% of the explained variance, was also significant (F_1,24_ = 21.10, *P* < 0.05) and positively related to the drainage and sanitary sewers densities (scores 0.33 and 0.31, respectively), while septic tanks density was negatively associated to this axis (score =  − 0.21). Phylogroups F/G and E, and cryptic clade IV and negatively related to RDA2, in contrast to phylogroups B2, D and B1.

Finally, partial RDA of spatial factors, constrained by environmental and urban infrastructure, showed that all the included AEMs (ranging from broad- to fine-scale resolution levels) were significant in explaining spatial variability (see Table S2 and Figure S2 in Appendix A2 of the Supplementary Material for more information).

## Discussion

Our study indicates that the spatial structuring of *E. coli* in a highly polluted urban stream network with favorable growth conditions is strongly influenced by the hydraulic and sanitary infrastructure and local ecological features of the habitat. The spatial distribution of *E. coli* abundance and the phylogenetic composition of its population reflect the complex interactions between the socio-technological attributes of the urban environment and the ecological conditions of the stream habitat, supporting our original hypothesis. We found that both urban infrastructure and micro-habitat conditions, which were in a straight relationship with spatial predictors, acted as important drivers of *E. coli* abundance in the streams network studied. Moreover, *E. coli* abundance was negatively correlated with phylogroup diversity. On the other hand, *E. coli* phylogenetic composition was driven by independent effects of spatial, infrastructure and environmental variables, as well as by a strong negative relationship between phylogroup diversity and dominance. We hypothesize that these results reflect the influence of distinct ecological processes on *E. coli* populations, thereby opening future avenues of research in other urban watersheds and additional hydro-climatic conditions.

It has been shown that the effects of geomorphological modifications made to urban streams (channel incisions, rectifications, concrete riverbanks, buried sections) result in a process of ecological simplification and in the predominance of exogenous control over internal equilibrium states, leading to undesirable resilient states^67,68^. In this vein, we hypothesize that the indivisible effect of infrastructure, environmental and spatial predictors explaining *E. coli* abundance patterns reflects the effect of large watershed-scale processes that cause a significant modification to local habitats, modulating microhabitat features that influence *E. coli* availability. Our hypothesis is supported by the negative co-variation observed between urbanization proxies (e.g., drainage density) and microhabitat features (e.g., macrophyte coverage).

Regarding the independent effects of environmental and urban conditions on *E. coli* abundance, we found a positive link with sanitary sewer density, which is in line with previous studies reporting the contribution of sewer leaks^68,69^. Furthermore, the association of *E. coli* abundance with macrophyte coverage suggests that its growth may be favored in locations with lower urbanization levels and aquatic vegetation. Indeed, macrophytes are known to be colonizable surfaces for *E. coli*^32^. Once established, and if nutrient levels are sufficient, bacteria may grow and eventually release cells into freshwater bodies. Surbeck et al.^8^ reported that *E. coli* grows above threshold concentrations of DOC (7 mg/L) and SRP (0.07 mg/L), evidencing its ability to exploit soluble nutrients available in urban freshwaters. The nutrient concentrations detected across the basin were above these values, thus providing favorable environmental conditions for growth. In addition, we found a significant correlation between DOC and the presence of *E. coli*, while other potential nutrients (DIN and Iron) were also spatially correlated with DOC. Altogether, these results validate our first prediction of a positive association of *E. coli* abundance with urban income sources and high nutrient availability.

We found several remarkable aspects in relation to phylogenetic composition. Phylogroups A and B1 showed a co-dominant structure throughout the sampled sites, suggesting their wide ubiquity regardless of differences in environmental conditions. This is in agreement with Petit et al.^16^ who reported co-dominance of A and B1 in the water column, with an increase in the frequencies of phylogroups A, D and F in urban locations. With respect to human gut microbiomes in South America, Stoppe^69^ found a predominance of phylogroup A, followed by B2, B1 and D, in an urban population from Brazil, in line with the results of Escobar-Páramo et al.^70^. On this basis, the dominance of the phylogroups detected in the stream network studied here suggests that human fecal contamination is widespread throughout the watershed.

In our study, lower frequency phylogroups (D, E, F/G and B2) were associated with water turbidity, which is positively correlated with water flow and the local density of sanitary sewers around each sampled site, and higher nutrient availability (e.g., SRP and DIN), an association that can be interpreted as a product of common sources of contamination. Our results also suggest that the occurrence of lower frequency phylogroups (B2, D, E, F/G) are mostly associated with recent external contributions from urban drainage infrastructures or septic tank exfiltration. This is consistent with the idea that some of them (such as D or F) occur in highly urbanized locations. On the other hand, B1 was linked to contrasting conditions where aquatic vegetation prevails, which is partially consistent with our second prediction, while there were no strong associations to urban infrastructures. Bearing in mind that B1 can be naturalized in water bodies -thus forming part of the indigenous microbiota- and that this phylogroup is able to colonize surfaces like macrophytes through biofilm development, our results may be interpreted as a positive environmental effect on B1 growth^18,19,71,72^. Finally, one strain belonging to the cryptic clade IV was isolated from a highly populated and urbanized location with low levels of sewerage infrastructure.

**Figure 6:**
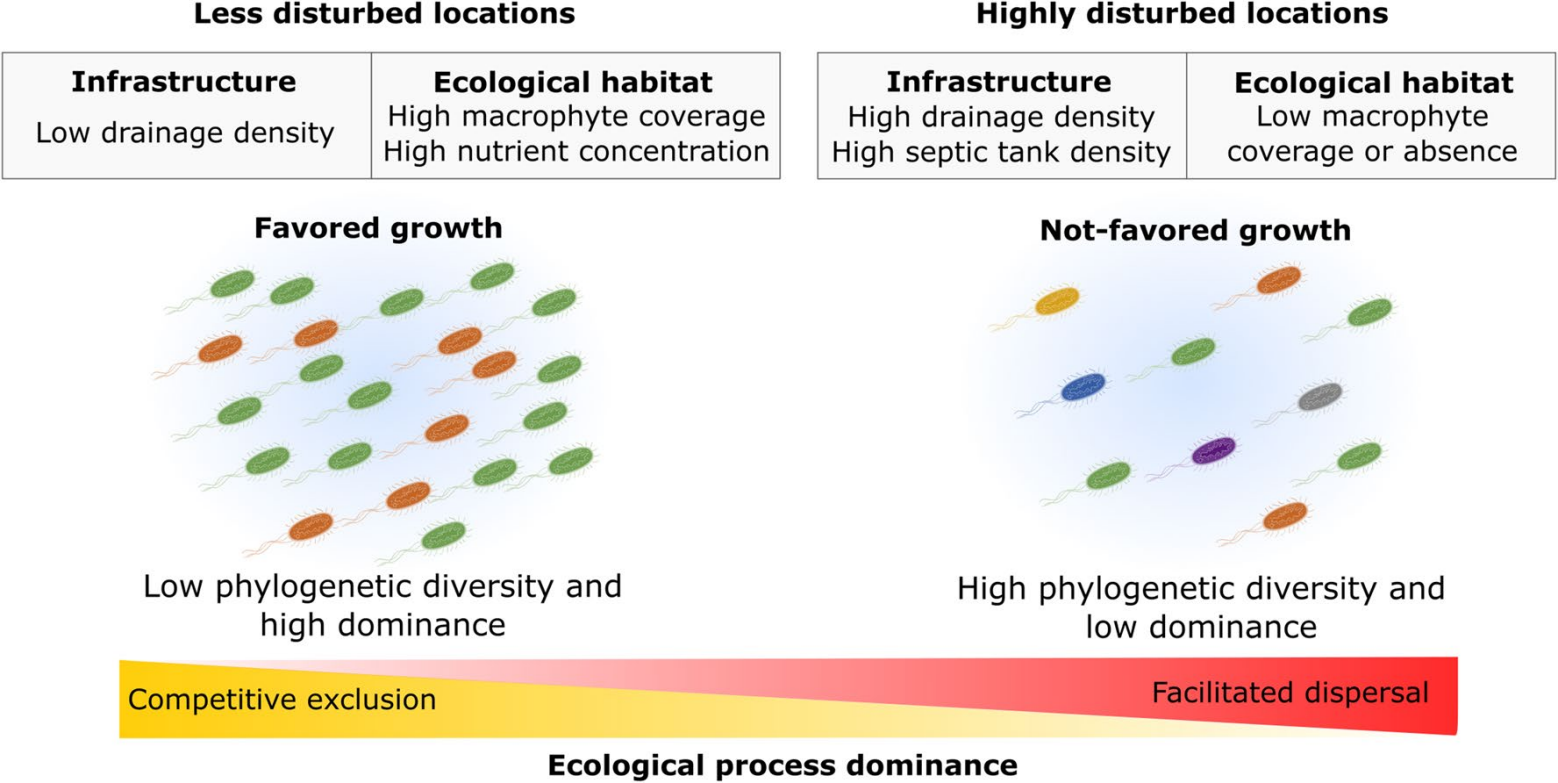
Hypothesized processes affecting the dynamics of *E. coli* population in an urban stream. Schematic diagram of contrasting scenarios along a gradient of environmental and urban conditions with different dominant ecological processes affecting the *E. coli* population. Graphic representation of *E. coli* from Togo picture gallery (Database Center for Life Science). https://togotv.dbcls.jp/en. Graphical edition in Inkscape V 0.92.4. Inkscape Project (2020). https://inkscape.org.

Altogether, our results can be interpreted in terms of different scenarios behind *E. coli* abundance and its phylogenetic composition in urban streams with favorable growth conditions (Fig. 6). A first scenario corresponds to locations with a relatively low level of disturbance, characterized by low infrastructure and stable habitat conditions, including the presence of high macrophyte coverage. These conditions, together with a high availability of nutrients, would promote high rates of bacterial growth and competitive exclusion of ecologically similar phylogroups. In this context, *E. coli* strains with the potential for survival and growth dominate the population structure, thus reducing the survival probability of lower frequency phylogroups. The second scenario involves highly disturbed locations with heavy external inputs. The lack of aquatic vegetation in local habitats and the periodic disturbances produced by drainage networks, may limit bacterial growth and offset competitive exclusion. The phylogenetic composition of *E. coli* populations in these habitats may be influenced by dispersal, facilitated through urban infrastructures and other diffuse sources like septic tanks leakage. Finally, the sanitary sewer network was also observed to positively contribute to the *E. coli* abundance, exerting its influence on *E. coli* occurrence irrespective of the environmental disturbance conditions.

Finally, we want to comment on some methodological issues of our analysis. First, we uncovered that the spatial predictors were relevant for explaining *E. coli* abundance and phylogenetic composition (Fig. 4). The selected broad- to fine-scale asymmetric eigenvectors reflect an expectable directional process through the stream network, involving a strong level of autocorrelation between close locations. They also reveal distinct spatially structured processes underlying transport-based (hydrological), spatial environmental or infrastructure patterns. It is worthy to note that the lack of updated information on public sanitary conditions and demographic characteristics may have introduced some bias into the selected AEMs showing pure spatial contributions. Therefore, the usage of spatial predictors allowed us to pull out spatial autocorrelation processes inherent to the structure of stream networks, enabling a more accurate analysis of the influence of local habitats or urban infrastructure features throughout the watershed. Second, the results and hypothetical mechanisms analyzed in this work must be assumed as general trends of the drivers for phylogroup diversity, since they arise from a single point in time, in favorable growth conditions (summer season). The use of presence/absence data due to limitations in sample size also implies a simplification of the real population structure, thus limiting further inferences. The analysis of the spatio-temporal dynamics of *E. coli* in urban several watersheds and the collection of a higher number of *E. coli* isolates will allow us to reach more extensive conclusions, while the present work offers contrastable hypothesis for further evaluation.

To conclude, our results provide information useful for global comparisons of *E. coli* persistence in the environment, while also having important implications for water quality assessment, as they question the use of *E. coli* as a proxy for recent microbiological pollution. In this regard, the genomic analysis of isolates, together with experimental assays may provide useful information on the determinants of *E. coli* environmental persistence and the differential fitness of phylogroups.

## Supplementary Information

Supplementary Information.
