# Supplementary material for: Unraveling the ecological processes modulating the population structure of Escherichia coli in a highly polluted urban stream network
Source: Sci Rep. 2021 Jul 19;11:14679. doi: 10.1038/s41598-021-94198-1 (PMC8289912; doi:10.1038/s41598-021-94198-1)
Supplement: Supplementary file 1 — Supplementary Information. [file 41598_2021_94198_MOESM1_ESM.docx]

**Supplementary Material**

**Unraveling the ecological processes modulating the population structure of *Escherichia coli* in a highly polluted urban stream network**

Martín Saraceno^1,2^, Sebastián Gómez Lugo^2^, Nicolás Ortiz^3^, Bárbara M. Gómez^3^, Carmen A. Sabio y García^2^, Nicolás Frankel^2,4^, Martín Graziano^1,2*^

^1^CONICET - Universidad de Buenos Aires. Instituto de Ecología, Genética y Evolución de Buenos Aires (IEGEBA). Buenos Aires 1428, Argentina. ^2^Universidad de Buenos Aires. Facultad de Ciencias Exactas y Naturales. Departamento de Ecología, Genética y Evolución. Buenos Aires 1428, Argentina. ^3^Instituto Nacional del Agua. Ezeiza 1804, Argentina. ^4^CONICET - Universidad de Buenos Aires. Instituto de Fisiología, Biología Molecular y Neurociencias (IFIBYNE). Buenos Aires 1428, Argentina. *Corresponding author: [marting@ege.fcen.uba.ar](mailto:marting@ege.fcen.uba.ar)

**Appendix A1: *E. coli* abundance and phylogroups assignation procedure results.**

**Table S1: By site *E. coli* mean abundance and phylogenetic groups absolute abundances**. *E. coli* abundance determination and phylogenetic annotation procedures are expressed in the “Methods and Materials” section. Results of *E. coli* abundance here presented are an average between the sub-sampling sites. Isolates for the phylogenetic annotation were picked randomly from sub-sampling sites plates. CCIV, cryptic clade IV; SE, standard error.

| Site | *E. coli* abundance (cfu/ml) | | A | B1 | B2 | D | E | F/G | CCIV | Isolates per site |
| --- | --- | --- | --- | --- | --- | --- | --- | --- | --- | --- |
|  | **Mean** | **SE** |  |  |  |  |  |  |  |  |
| LP1 | 108 | 27.1 | 19 | 1 | 0 | 1 | 0 | 0 | 0 | 21 |
| LP2 | 3 | 1.7 | - | - | - | - | - | - | - | - |
| LP3 | 6100 | 513.3 | 15 | 7 | 0 | 2 | 0 | 1 | 0 | 25 |
| LP4 | 2320 | 666.3 | 12 | 6 | 1 | 0 | 0 | 0 | 0 | 19 |
| LP5 | 2675 | 1723.4 | 13 | 5 | 0 | 5 | 0 | 1 | 0 | 24 |
| LP6 | 736 | 232.1 | 24 | 1 | 0 | 0 | 3 | 0 | 0 | 28 |
| SF1 | 767 | 384.5 | 11 | 3 | 1 | 2 | 1 | 2 | 0 | 20 |
| SF2 | 10525 | 3739.5 | 33 | 0 | 0 | 0 | 0 | 0 | 0 | 33 |
| SF3 | 188 | 27.7 | 7 | 7 | 1 | 1 | 1 | 1 | 0 | 18 |
| SF4 | 145 | 85.4 | 13 | 3 | 0 | 1 | 1 | 6 | 0 | 24 |
| SF5 | 175 | 64.7 | 14 | 3 | 1 | 5 | 0 | 4 | 1 | 28 |
| SF6 | 899 | 564.1 | 14 | 2 | 1 | 8 | 2 | 1 | 0 | 28 |
| SD1 | 10700 | 3511.0 | 15 | 9 | 0 | 3 | 2 | 0 | 0 | 29 |
| SD2 | 2950 | 750.0 | 19 | 5 | 2 | 4 | 0 | 0 | 0 | 30 |
| **Isolates per phylogenetic group** | | | 209 | 52 | 7 | 32 | 10 | 16 | 1 | Total: 327 |

**Appendix A2: Spatial predictors and supplementary results to pRDA analyzes.**

**Figure S1**: **Maps of selected Asymmetric Eigenvector Maps (AEMs) for positive and negative spatial autocorrelation.** AEMs were selected through a *forward selection* procedure associated to each response variable. In the case of *E. coli* phylogroups occurrence, a subset of selected AEMs with the greater R^2^ contribution was employed in the variance partition analysis. AEMs used for each response variable analysis are identified with a square symbol next to each AEM label (*E. coli* abundance), and a star symbol (*E. coli* phylogroups occurrence). Significant AEMs in pRDA analysis are indicated in fuchsia.

**
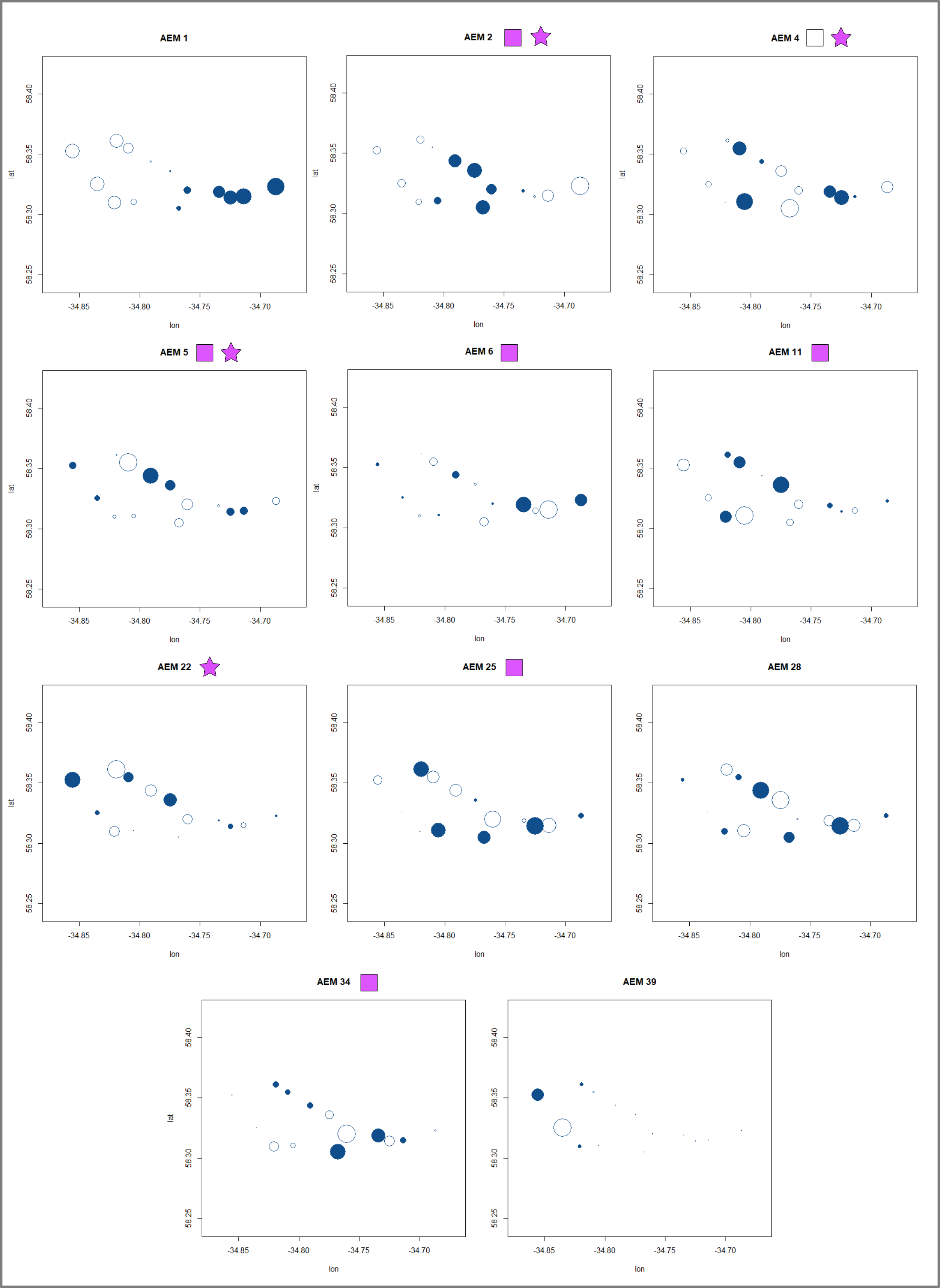
**

**Table S2: Partial RDA analysis of pure effects of spatial matrix.** The contribution of each set of spatial predictors was assessed for each of the response variables through a pRDA analysis conditioned by environmental and urban infrastructure sets. A) *E. coli* abundance as response variable; B) **Occurrence of *E. coli* phylogroups** as response variable. DIN, dissolved inorganic nitrogen; SRP, soluble reactive phosphorous; AEM, asymmetric eigenvector map.

| **A)** | **F value** | **Significance** (P<0.05) |
| --- | --- | --- |
| Spatial predictors matrix  (global model) | F_7,24_= 11.94 | * |
| AEM2 | F_1,24_= 6.37 | * |
| AEM4 | F_1,24_= 3.56 |  |
| AEM5 | F_1,24_= 5.57 | * |
| AEM6 | F_1,24_= 9.13 | * |
| AEM11 | F_1,24_= 6.36 | * |
| AEM25 | F_1,24_= 8.26 | * |
| AEM34 | F_1,24_= 5.19 | * |
| RDA1 axis | F_1,30_= 104.46 | * |
| **B)** | **F value** | **Significance** (P<0.05) |
| Spatial predictors matrix  (global model) | F_4,24_= 63.50 | * |
| AEM2 | F_1,24_= 23.63 | * |
| AEM4 | F_1,24_= 79.72 | * |
| AEM5 | F_1,24_= 25.89 | * |
| AEM22 | F_1,24_= 4.69 | * |
| RDA1 axis | F_1,24_= 198.37 | * |
| RDA2 axis | F_1,24_= 42.51 | * |

**Figure S2:** **Effects of spatial predictors on the occurrence of *E. coli* phylogroups**. Biplot of pRDA analysis of AEMs controlling for the rest of the variable sets (scaling = 3). Significant variables are identified with an asterisk. AEM, asymmetric eigenvector map.


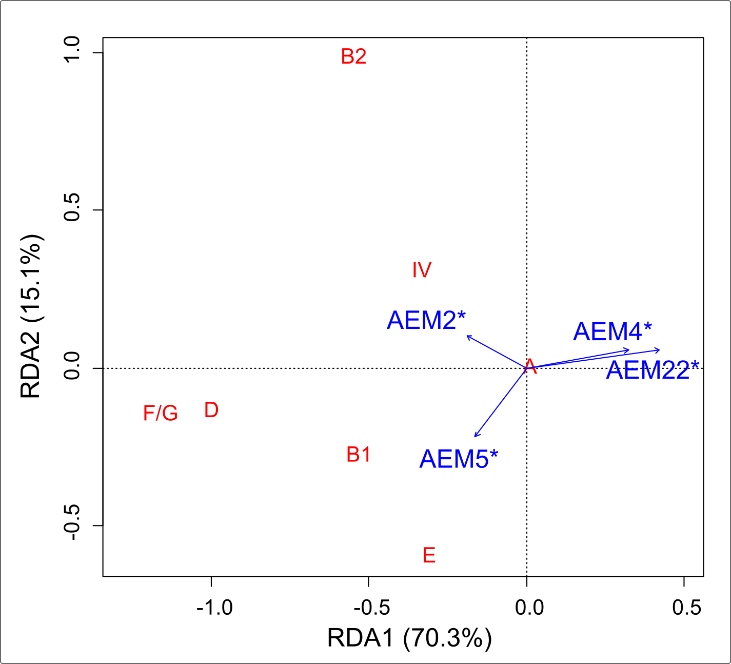


**Appendix A3: Variable selection procedure**

A principal component analysis (PCA) was performed separately for each predictor set of environmental and urban infrastructure, and groups of covariate variables were graphically identified. Cumulative variance proportion explained by first two PCA axes was 60% for environmental variables and 90% for urban variables (Supplementary Figure S3). Based on the PCA results and the analysis of correlations between all available variables, we selected variables for variance partitioning analysis across each covariate group, also considering the ecological relevance of each variable based on previous knowledge related with the persistence of *E. coli* in secondary habitats. Among the environmental set of variables, we retained parameters related to relevant nutrients such as DIN, SRP, Iron and DOC, and variables affecting the physical availability of bacteria in the water column, such as flow velocity and mean water column depth. Moreover, macrophyte coverage and turbidity were also selected, as the association of bacteria to surfaces through biofilm formation might play an important role on its availability in the water column. Parameters with low spatial variability or with known high temporal daily variations such as pH, OD and temperature were not selected. Within urban predictors, it was observed a high correlation cluster of variables between population and road densities, and the impervious surface coverage, selecting the impervious surface coverage as it was the less correlated parameter with the rest of the infrastructure parameters (Spearman’s rho and VIF estimators). Additionally, drainage density was selected against drinking water coverage, due to its hypothesized association with bacterial entry to streams. Finally, a PCA analysis and the variance inflation factor (VIF) was computed into the final predictor sets to assure an appropriate proportion of explained variance as well as to control for excessive collinearity (see Supplementary Table S3 for VIF values).

**Figure S3: Results of principal component analysis (PCA) including all available variables within the environmental set (right) and the urban set (left).** Graphs represent biplots (type 3 scaling). DIN, dissolved inorganic nitrogen; SRP, soluble reactive phosphorous, DOC, dissolved organic carbon; DO, dissolved oxygen.

**
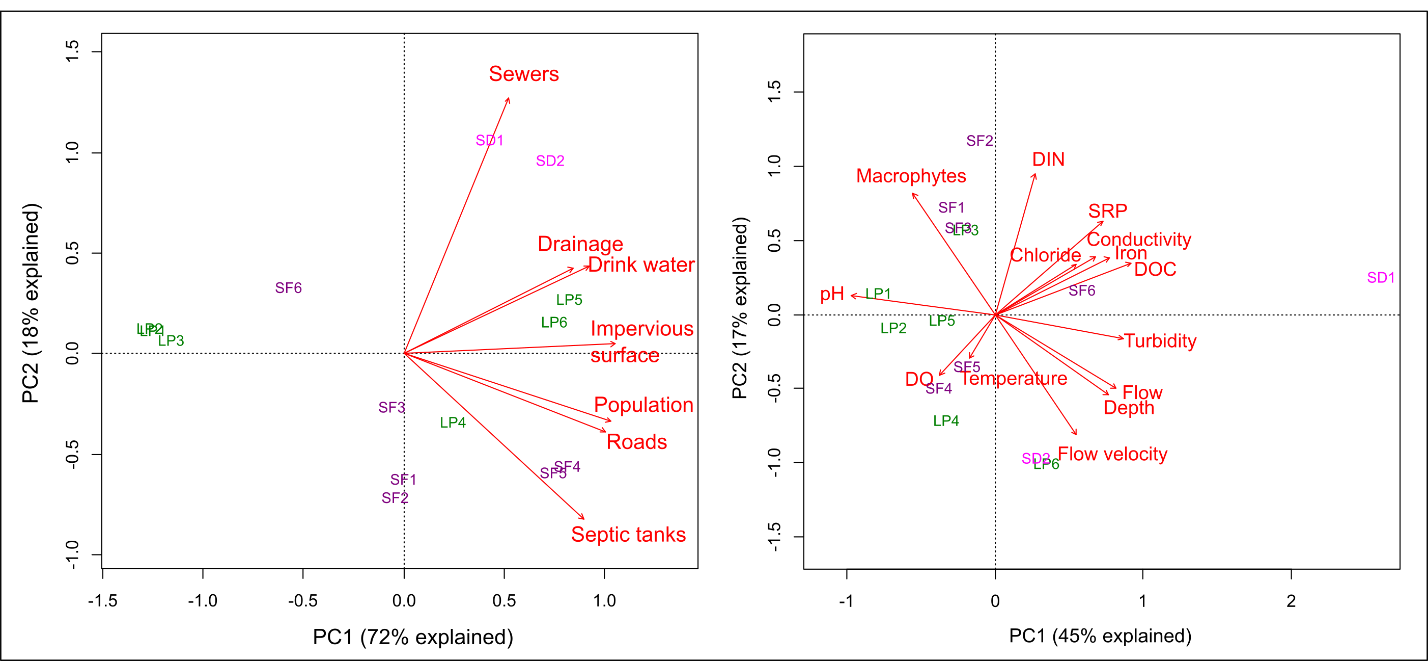
**

**Table S3: Variance inflation factor (VIF) values for each selected predictor set.** Differences between response variables are due to the different number of samples used for analysis (LP2 was discarded for phylogenetic analysis due to the lack of available isolates). Environmental parameters are unshaded, while urbans are shaded in light gray. DIN, dissolved inorganic nitrogen; SRP, soluble reactive phosphorous.

| **VIF** | | |
| --- | --- | --- |
|  | *E. coli* abundance | *E. coli* phylogenetic composition |
| Macrophyte coverage | 2.32 | 2.04 |
| DIN | 3.20 | 3.99 |
| SRP | 4.37 | 5.71 |
| Turbidity | 2.95 | 2.71 |
| Iron | 1.65 | 1.86 |
| Water Depth | 2.75 | 2.48 |
| Flow velocity | 2.27 | 2.66 |
| Sanitary sewer density | 1.54 | 1.51 |
| Septic tanks density | 1.46 | 1.36 |
| Drainage density | 1.96 | 1.73 |

**Appendix A4: Supplementary information on the global correlations between variables.**

**Table S4: Results of node centrality statistics.** A correlation matrix of all variables was obtained through Pearson’s method. Then, it was employed as input to establish a correlations network including only the correlations among variables greater than 0.5. The Betweenness estimator reflect the number of interacting nodes for each variable, and the Expected Influence estimator the degree of interaction, considering the number of positive and negative interacting nodes weighted by the correlation between each variable. DO, dissolved oxygen; DIN, dissolved inorganic nitrogen; SRP, soluble reactive phosphorous; DOC, dissolved organic carbon.

|  | **Betweenness** | **Expected Influence** |
| --- | --- | --- |
| pH | 21 | -4.817574384 |
| Conductivity | 17 | 2.538391980 |
| Temperature | 0 | 0.510314928 |
| DO | 0 | 0.510314928 |
| DIN | 7 | -0.005191183 |
| SRP | 7 | 2.439327046 |
| DOC | 10 | 3.599434141 |
| Turbidity | 0 | 1.353034908 |
| Chloride | 6 | 1.523783836 |
| Iron | 0 | 1.554592377 |
| Macrophytes coverage | 2 | -1.907411653 |
| Mean flow | 1 | 2.457246490 |
| Mean flow velocity | 6 | 2.483385819 |
| Depth | 1 | 1.359467538 |
| Drinking water coverage | 13 | 3.650032514 |
| Sanitary sewer density | 12 | 2.946458441 |
| Septic tanks density | 0 | 2.333566994 |
| Impervious surface | 34 | 4.797111509 |
| Drainage density | 10 | 1.891505510 |
| Roads density | 0 | 2.950515938 |
| Population density | 2 | 3.447489698 |
| *E. coli* | 0 | 1.461723305 |

**Figure S4: Relationship of *E. coli* abundance with environmental and urban infrastructure features.** Clustered correlogram of environmental and urban infrastructure parameters. Levels of significance of Pearson’s correlation among parameters are expressed as ‘***’, p<0.0001; ‘**’, p<0.001; ‘*’ and p<0.05.

**
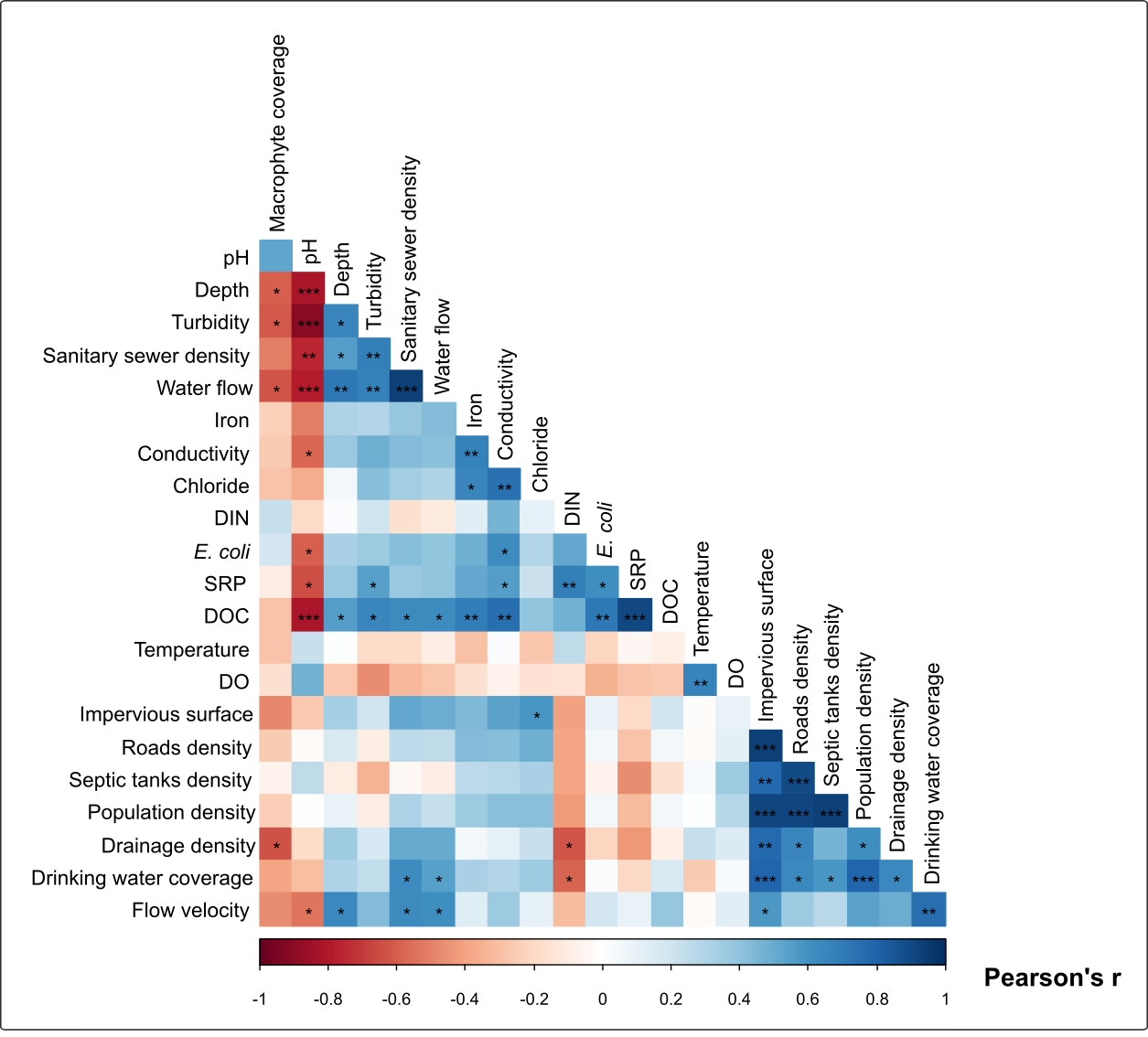
**
